# Interactions of Bacteriophages and Bacteria at the Airway Mucosa: New Insights Into the Pathophysiology of Asthma

**DOI:** 10.3389/falgy.2020.617240

**Published:** 2021-01-26

**Authors:** Panagiota Tzani-Tzanopoulou, Dimitrios Skliros, Spyridon Megremis, Paraskevi Xepapadaki, Evangelos Andreakos, Nina Chanishvili, Emmanouil Flemetakis, Grigoris Kaltsas, Styliani Taka, Evangelia Lebessi, Anastassios Doudoulakakis, Nikolaos G. Papadopoulos

**Affiliations:** ^1^Allergy and Clinical Immunology Unit, 2nd Pediatric Clinic, National and Kapodistrian University of Athens, Athens, Greece; ^2^Laboratory of Molecular Biology, Department of Biotechnology, School of Food, Biotechnology and Development, Agricultural University of Athens, Athens, Greece; ^3^Division of Evolution and Genomic Sciences, University of Manchester, Manchester, United Kingdom; ^4^Center for Clinical, Experimental Surgery and Translational Research of the Biomedical Research Foundation of the Academy of Athens, Athens, Greece; ^5^Laboratory for Genetics of Microorganisms and Bacteriophages, Eliava Institute of Bacteriophage, Microbiology & Virology, Tbilisi, GA, United States; ^6^Department of Electrical and Electronic Engineering, University of West Attica, Athens, Greece; ^7^Department of Microbiology, P. & A. Kyriakou Children's Hospital, Athens, Greece

**Keywords:** bacteria, asthma, bacteriophages, airway mucosa, tripartite symbiosis

## Abstract

The airway epithelium is the primary site where inhaled and resident microbiota interacts between themselves and the host, potentially playing an important role on allergic asthma development and pathophysiology. With the advent of culture independent molecular techniques and high throughput technologies, the complex composition and diversity of bacterial communities of the airways has been well-documented and the notion of the lungs' sterility definitively rejected. Recent studies indicate that the microbial composition of the asthmatic airways across the spectrum of disease severity, differ significantly compared with healthy individuals. In parallel, a growing body of evidence suggests that bacterial viruses (bacteriophages or simply phages), regulating bacterial populations, are present in almost every niche of the human body and can also interact directly with the eukaryotic cells. The triptych of airway epithelial cells, bacterial symbionts and resident phages should be considered as a functional and interdependent unit with direct implications on the respiratory and overall homeostasis. While the role of epithelial cells in asthma pathophysiology is well-established, the tripartite interactions between epithelial cells, bacteria and phages should be scrutinized, both to better understand asthma as a system disorder and to explore potential interventions.

## Introduction

Asthma is a chronic inflammatory disease presenting with wheeze, chest tightness and shortness of breath; however these symptoms may vary in appearance, frequency and intensity (1). Underlying mechanisms include genetic and environmental factors acting already from pregnancy and during the first years of life (2, 3), although new cases may appear at any age (4). It is generally accepted that the term “asthma” incorporates a variety of clinical and mechanistic entities (5, 6). Terms such as phenotypes, endotypes, subtypes or clusters are frequently used to subdivide and classify asthma, without however conclusive consensus. Affecting almost 300 million people worldwide, including up to 10% of children (7, 8), asthma has emerged as a significant health problem with huge economic implications on healthcare systems globally (9, 10).

Allergic sensitization of the host and subsequent overreaction to common environmental factors with typical inflammatory and immunological responses are strong determinants for the onset and the development of asthma. The airway epithelium is the primary interphase between inhaled triggers and the host, playing a central role on asthma pathophysiology (development, progression, and exacerbation). The epithelium of the large and small airways of the lungs is constituted by basal and undifferentiated columnar cells as well as ciliated and secretory (large airways) or Clara cells (small airways), forming pseudostratified or columnar and cuboidal structures, respectively. Additionally, distal alveoli are lined by alveolar type I and II epithelial cells (11). The whole spectrum of epithelial lining across the respiratory tract is a physical “tight-sealed” protective barrier against external stressors having also multiple regulating and signaling properties. The epithelial barrier integrity is ensured mainly by the formation of intracellular tight junctions (TJs) and adhesions and secondly by the presence of mucus and periciliary glycocalyx layers produced by adjacent secretory cells. This physical barrier ensures the elimination of noxious agents and their rejection through mucociliary clearance (MCC). This first line of innate immune defense protects the airway tract from incoming pathogens such as bacteria and viruses, as well as other chemical and physical stressors that can act as allergens in the case of atopic asthma.

The asthmatic epithelium has structural and functional abnormalities, probably due to genetic predisposition (12) giving expression of, among other, an “over-reaction” to common environmental allergens. Being under repetitive injury and inflammation, the asthmatic epithelium progressively remodels its structural characteristics (13). Specific allergens or pathogens can induce dysfunction and/or disruption (14–17) of the TJs of the cells and access the airway submucosa through the paracellular way, facilitating sensitization against them. Its demonstrative that *in-vitro* infection of airway epithelial cells, obtained from children with or without asthma, with human rhinovirus, provokes different expression of TJs proteins. In the case of asthmatic cells it was observed permanent reduction of TJs expression and impairment of epithelial barrier integrity (18). In parallel, changes on the mucus composition and production rates in asthmatics have great impact on the MCC (19, 20). The asthmatic epithelium become more susceptible to infections (21), secreting a cascade of chemokines and cytokines which in turn activate several immune cells (22, 23). In the case of airway epithelial cells, obtained from asthmatic children and infected with human rhinovirus in Air Liquid Interfase cultures (ALIs), it was observed reduced IFN-β and increased inflammatory cytokine production, compared to healthy control (24). Activation of immune cells due to persistent allergic inflammation in asthma progressively alters the bronchial physiology into a pathological state where airway smooth muscle cells hyperplasia, subepithelial fibrosis, extracellular matrix overproduction, continuous angiogenesis, and mucus hypersecretion are detected (25). As a consequence, bronchial wall inflammation and thickening cause airway obstruction resulting in the typical respiratory symptoms of asthma. Strikingly, it has been suggested that epithelial mediation on allergic and immune responses has an impact on epithelial cell memory, establishing a type of epigenetic imprint on them (21).

The human microbiome is a dynamic and diverse entity, composed of an array of microbial genomes from bacteria, phages, fungi, protozoa and viruses. It is affected by immigration, elimination and growth of microbes within and possibly between different niches of each individual (26–28). Despite existing variability, healthy microbial populations share a few common characteristics and abundances that change significantly in the cases of chronic inflammatory diseases, such as asthma (29)where microbial imbalance or “dysbiosis” occurs (30, 31). Indicatively, it is reported that the airways of healthy individuals are dominated mainly by bacteria of the genera *Prevotella* (*Prevotella melaninogenica, Prevotella nanceiensis, Prevotella salivae*) and *Veilonella* (*Veillonella alcalescens, Veillonella parvula, Veillonella dispar*) (26, 32–34). In contrast, the bacteriome of asthmatic airways is dominated by *Haemophilus* (*Haemophilus influenzae* B, non-typable *Haemophilus influenzae*), *Neisseria* and *Moraxella catarrhalis* along with *Streptococcus pneumoniae* and *Staphylococcus aureus* (32, 35). Domination of specific bacterial species in asthma raises several questions about a potentially causal role of microbiome imbalance to this disorder (36, 37) and the role of epithelial defense to microbial attachment and invasion (38–41).

Maintenance of the microbial equilibrium/homeostasis is influenced not only by epithelial responses and host immunity, but also and possibly to a greater extent, by bacterial viruses that colonize, along with bacteria, human mucosal surfaces. Phages are capable of controlling microbial populations, expressing high genetic variation and complexity. They are known as the most abundant biological entities on the planet (42) with many references for their presence in the human body and their direct interactions with eukaryotic cells, organs and tissues (43–45). There is a plethora of lytic phages that target bacterial populations, indirectly protecting epithelial surfaces from bacteria colonization and/or overpopulation. On the other hand, there are phages following mainly a lysogenic life cycle integrating their genome on bacterial chromosomes and remaining as prophages in a lethargic state. Induction of prophages may be related with the acquisition of bacterial DNA which includes toxin or antibiotic resistance related genes. Horizontal transfer of these genes (HGT) between bacteria may have an impact on bacterial population fitness across the human body (46–48). The human “phageome” imposes important selective pressure on bacterial populations, influencing and determining along with the eukaryotic host the overall health and well-being (49). It seems that microbe-phage interactions along with epithelium responses participate in a co-evolving game of symbiotic and/or antagonistic relationships thus their simultaneous study could add insight into the pathophysiology of asthma.

Within the context of this review we explore the importance of the triptych: phage-bacterium-respiratory epithelium, toward asthma pathogenicity and development. The current literature around this complex biological system is limited and focused mainly on the gut epithelium and its interactions with local bacteria and phages. Undoubtedly, the information gap around asthma development and the contribution of the triptych remains unbridged. We first describe the current understanding about the role of microbiome in asthma development and exacerbation and then assess the interactions of specific bacterial species with the respiratory epithelium. We further elucidate the direct interactions between phages and human epithelial cells and finally identify the few existent *in-vitro* and *in-vivo* studies that have assessed the role of the triptych in respiratory disease models.

## Subsections

### The Microbiome in Asthma

Healthy adults are breathing ~7000L of air per day (50, 51) and their upper and lower airways are constantly exposed to a variety and large number of microorganisms. The estimated load of inhaled bacteria, viruses and fungi ranges between thousands to millions of particles per day, with the exact number depending upon the environmental exposure (52–54). The nasal cavity, pharynx and paranasal sinuses are part of the upper respiratory tract and their microbial communities along with the oral microbiome determine in a decisive way the lower respiratory tract microbial composition in healthy states (55, 56). The proposed “Adapted Island Model of Lung Biogeography” describes effectively the immigration rates of microbial communities from the upper respiratory tract to the large and small airways and the distal alveoli of the lungs (26, 57). It is assumed that the respiratory tract should be considered as a large and unique ecosystem with different environmental niches affecting decisively the immigration rates and the biodiversity levels of microbial communities in healthy states. Undoubtedly, the whole identity of the airways microbiota is drastically changing in the cases of respiratory diseases such as asthma and the overall physiology and airway function is affected (58–60). Therefore, the delineation of microbial identities in health and asthma could lay new perspectives for future phenotyping and medical interventions.

The microbiome in the gut may also have a role in asthma pathogenesis by modulating the immune system via the induction of immune cells that circulate and reach the lungs, regulating and influencing lung immunity and microbial equilibrium from the first years of life (27, 61–64). The recently emerged lung-gut axis hypothesis was proposed by Schuijt TJ et al. (65), showing that healthy gut microbiota efficiently protect mice lungs from *S. pneumoniae* infection through the activation of alveolar macrophages and enhanced levels of immunomodulatory cytokines (65). The exact mechanisms by which the gut-lung axis activates innate immune system in health and disease are still unknown. Nevertheless, interactions between respiratory microbiota and the airway mucosa are arguably at least as important in shaping local immunity and dysbiosis in asthma.

In the last decade, culture independent molecular techniques, mostly based on sequencing of the hypervariable regions of the 16S rRNA gene have been used to characterize the airway microbiome in conditions of health and disease (26, 66, 67). It was shown that the bronchial tree is colonized across its whole spectrum by specific microbial phyla thus having its own microbial identity and being protected from infections and chronic respiratory disease development. Hilty et al. analyzed the airway microbiota of the nose, oropharynx and lungs in asthmatic and chronic obstructive pulmonary disease (COPD) patients and compared the results with healthy individuals (35). Using nasal and oropharyngeal swabs as well as bronchoscopic cytology brushings and broncho-alveolar lavage fluid, they performed cladistic analysis to identify the distribution and abundance of airway microbiota across the spectrum of health and disease. They concluded that pathogenic Proteobacteria, particularly with *Haemophilus, Neisseria*, and *Moraxella* genera were more frequent in airways of asthmatic and COPD patients compared to healthy individuals and maybe related to increased risk of early asthma development when found in infant's pharynxes. In addition, *Staphylococcus* and *Streptococcus* dominance was a common characteristic in children with refractory asthma, while in healthy airways, *Prevotella* and *Veillonella* were among the prevalent genera (35). Microbial profiles in sputum samples from severe asthmatics were also identified and compared with healthy individuals in another study. The prevalent bacteria in severe asthmatics were amongst the genera of *Haemophilus* or *Streptococcus* and *M. catarrhalis* species (68). Prevalence of specific bacterial species in the respiratory tract of asthmatics compared to healthy controls seems to reduce the microbial diversity and affect in a decisive yet underexplored way the pathophysiology in asthma.

Observations from wider population studies were generally confirmatory and tried to connect early microbiome fluctuations with asthma development in childhood. A Danish birth cohort of 700 children followed until the age of 6 years showed that airway microbiota dominated by *S. pneumoniae* and subsequent immune responses were correlated with asthma development risk (69, 70). In another study, nasal samples from 6 to 12 years old asthmatic children had reduced microbiota diversity compared to healthy controls and a notably high abundance of the genus *Moraxella*. The question raised was whether the loss of abundance in asthmatics is due to prevalence of Moraxella (71). It has been suggested that *Moraxella* species coming from the upper respiratory system of asthmatic patients are also identified in abundance at the lower airways (72, 73). In another study, infant's nasopharynxes were screened during the first year of life where colonization from common respiratory pathogens occurs. It was proposed that early colonization of nasopharynxes with *Moraxella, Staphylococcus, Haemophilus*, and/or *Streptococcus* can provoke upper respiratory tract infections and inflammation that possibly spread to the lower airways, predisposing for future asthma onset in childhood (74, 75). It is apparent that the neonatal airway microbiome in conjunction with genetic predisposition, tissue impairment and/or respiratory infections may act causatively in regard to asthma development from the first years of life and into adulthood. In Table 1 we summarize important clinical studies about the microbiome profile in asthmatic children populations and the role of specific bacterial species found to dominate in their airways.

**Table 1:** Studies of the microbiome profiles of asthmatic infants and children and the assessment of the role of each prevalent bacterium in asthma disease.

**References**	**Population**	**Respiratory niche**	**Prevalent bacteria**	**Outcomes**
Thorsen et al. (70)	700 infants from asthma history families	Hypopharynx, Lungs	*Staphylococcus, Streptococcus, Moraxella, Haemophilus, Corynebacterium*	Risk for asthma development in the first 6 years of life
Bisgaard et al. (69)	321 neonates from asthma history families	Hypopharynx	*Moraxella catarrhalis, Haemophilus influenzae, Streptococcus pneumoniae*	Recurrent wheeze, asthma, and allergy risk in the first 5 years of life
Depner et al. (71)	68 asthmatic children	Nasopharynx	*Moraxella*	Loss of microbial abundance due to the prevalence of *Moraxella*
Teo et al. (75)	234 infants from asthmatic mothers	Nasopharynx	*Moraxella, Haemophilus, Streptococcus*	Development of acute upper respiratory tract infections, atopy and wheeze
Davis et al. (74)	16 asthmatic children and adults	Nasopharynx	*Staphylococcus aureus*	Increased risk of asthma prevalence, symptoms, and exacerbations in children and young adults
McCauley et al. (76)	413 asthmatic children	Nasopharynx	*Moraxella catarrhalis*	Increased risk for asthma exacerbation and eosinophil activation
McCauley et al. (76)	413 asthmatic children	Nasopharynx	*Corynebacerium, Staphylococcus aureus*	Reduced respiratory illness and exacerbation events
McCauley et al. (76)	413 asthmatic children	Nasopharynx	*Streptococcus pneumoniae*	Increased risk of rhinovirus infection
Perez-Losada et al. (77)	40 asthmatic children and adults	Nasopharynx	*Moraxella, Staphylococcus*	Provoke upper and lower airways infections in asthmatics
Kloepfer et al. (78)	166 asthmatic children	Nasopharynx	*Streptococcus pneumoniae, Moraxella catarrhalis*	Contribute to the severity of respiratory tract illnesses, including asthma exacerbations
Yin et al. (79)	139 asthmatic children	Serum	*Mycoplasma pneumoniae*	High levels of IgM and eosinophils

In respect to asthma exacerbations, nasopharyngeal microbiota dominated by *M. catarrhalis* and *S. aureus* in children with asthma were associated with increased risk of asthma exacerbation and eosinophil activation (76, 77). Moreover, in the course of a rhinovirus infection, *M. catarrhalis* and/or *S. pneumoniae* were prevalent in the nasal samples of children with or without asthma, contributing to asthma exacerbations (78). Bacterial diversity possibly constitutes a major factor for the maintenance of the physiologic function of the airways and the control of asthma inflammation (59). Studies in patients with neutrophilic asthma (80) or mild asthma (81) advocate that reduced bacterial diversity is associated with neutrophilic inflammation and bronchial hyper responsiveness, respectively. In another study, it was shown that prevalence of *H. influenzae, Pseudomonas aeruginosa* and *S. aureus* at the lower airways of severe asthmatics was associated with persistence of asthma and exacerbation events but not with bronchial remodeling (82). *P. aeruginosa*, isolated from sputum of asthmatic patients was also found to be related with exacerbation (83).

It is reported from various *in-vivo* studies that *Mycoplasma pneumoniae* can affect the cilia structure of the respiratory epithelium and promote chronic inflammation, smooth muscle contraction, cytokine and antibody production (84–86), typical reactions during asthma onset and exacerbation (87, 88). *M. pneumoniae* infection in asthmatic children it was also connected with more IgM production and eosinophils in the blood compared to healthy controls and with, increased susceptibility to asthma symptoms (79). A meta-analysis from Zehua L. et al., confirm the possible implication of *M. pneumoniae* infections with asthma (79). Several serological studies also suggest that *Chlamydia pneumoniae* is related with severity phenotypes in asthma (89–91). Although the complexity of the microbiome is high, most of the studies suggest that Proteobacteria more than Firmicutes prevail and are common among the microbiota of patients with lung diseases (92), such as asthma or cystic fibrosis (93). It is therefore plausible that inflammatory signals resulting from such colonization may play an important role in persistent inflammation in asthma.

### The Complex Dynamics of Bacteria- Epithelium Interactions in Asthma

*Moraxella catarrhalis* and *Haemophilus influenzae* are gram-negative opportunistic pathogens carried as commensals in the respiratory tract. Gram-negative bacteria predominate within the respiratory tract (94) and are mostly colonizing asymptomatically, although they can become causative agents for childhood otitis and other respiratory infections, such as pharyngitis and pneumonia (95). Moreover, they have been implicated in exacerbations of asthma and COPD (96, 97). *S. pneumoniae* and *S. aureus are* gram-positive extracellular bacteria colonizing mucosal surfaces of the upper respiratory tract from the early childhood. Infection from *S. pneumoniae* ranges from asymptomatic pharyngeal carriage and typical mucosal diseases like otitis media and sinusitis up to severe invasive diseases such as pneumonia, endocarditis, meningitis and bacteremia (98). *S. aureus* infections are also characterized by initial nasal colonization that may lead to subsequent invasive disease of skin or internal organs (99). The microbial identity of the human nasopharynx is unique for each individuals occupation with different serotypes of each species and seasonal modifications are influencing in a decisive way the overall image (100). It has been shown that microbial interactions are affected and change in the context of a respiratory disease (101) where the causative microbe prevail against others (102) and subsequent immune responses of the host alter the environment for all the resident bacteria (103). In asthma, chronic inflammation creates a favorable environment for transmission and colonization of airway bacteria to different respiratory niches (104). It is thus important to understand the dynamics of bacterial colonization and the events of adhesion and invasion to epithelial cells, as the possibility of intervention.

The evaluation of virulence or commensalism nature of *M. catarrhalis* and *H. influenzae* can be deemed according to several bacterial components (105), in accordance to their adherence capacity to epithelial surfaces (106) and their resistance to the complement cascade activation. The most significant surface components of *M. catarrhalis* and *H. influenzae* belong to the outer membrane proteins (OMP) and lipooligosaccharides (LOS). Serotypes of *S. pneumoniae* and *S. aureus* strains are mainly distinguished according to capsular polysaccharides on bacterial isolates that give different antigenicity and immunological reactions (107, 108). *S. pneumoniae* is classified into >90 different serotypes carrying different chemical identities on their cell wall and expressing distinct specificities across epithelial cells (109, 110). *S. pneumoniae* serotypes have been used during the last decades for the development of effective vaccination programs in children for the prevention of respiratory diseases caused by this pathogen (94). The abundance of serotypes underpins the complex relationships amongst species and the spectrum of their virulence across epithelial surfaces.

OMPs induce the release of pro-inflammatory cytokines and chemokines, like interleukin 1b (IL-1b), IL-6, IL-8 and prostaglandin E2 that promote adhesion of bacteria to the epithelium. LOS is a major trigger of the inflammatory response and plays a role in both adherence and invasion to the host cells (111, 112). LOS is an outer membrane glycolipid, involved in both adherence (113, 114) and invasion (115) to epithelial cells and in serum resistance (114). In several population studies three differentially distributed serotypes of *M. catarrhalis* according to LOS type (60–75% A, 20–30% B and 2–6% C) were identified (116–118). However, from these studies there is no clear correlation between serotypes and *M. catarrhalis* infection events. Nevertheless, a higher frequency of LOS type B and a lower frequency of LOS type A in adults compared to children was observed (117). *H. influenzae* isolates can be divided into encapsulated (serotypes a-f) and non-encapsulated strains designated also as typable or non-typable isolates accordingly. Colonization of nasopharynx with typable b serotype (<3% of population) causes, in most of the cases, serious invasive disease such as meningitis (119, 120), while colonization with non-typable serotypes (80% of adults and 40% of children) may result in sinusitis, exacerbation of COPD, conjunctivitis and otitis media (121).

Several human cell lines have been utilized to assess adherence and invasion events *in-vitro*, including A549 (type II pneumocytes), HEp-2 (laryngeal), Chang (conjuctival), Detroit 562 (pharyngeal), NCIH 292 (lung epidermoid), BEAS-2B (bronchial) and 16HBE14o (bronchial) cells (122–124). Recent *in-vitro* studies suggest that infection with *M. catarrhalis* provokes more cellular damage and inflammation (IL-33 and IL-8 production) compared to *S. aureus* (76, 125). In an *in-vitro* study, A549 were infected with *M. catarrhalis* and the presence of NOD1 and toll-like receptors (TLR2) were found to play a significant role in bacterial invasion and subsequent IL-8 production (126). In another *in-vitro* study they examined by electron and confocal microscopy the invasion capacity of *M. catarrhalis* in BEAS-2B cells, A549 and primary small airway epithelial cells. Formation of lamellipodia and filopodia from the infected epithelium toward *M. catarrhalis* after adherence was demonstrated, suggesting a macropinocytosis-like mechanism of invasion that comprises a PI3K-dependent contractile mechanism that transforms phagosomes and ruffles into closed intracellular organelles. Bacterial invasion increased steadily up to 6 h after the addition of bacteria and they calculated that ~5% of all infected BEAS-2B were invaded by *M. catarrhalis*. This suggests that invasion of this extracellular bacterium may be more common than previously thought. Interestingly, an *ex-vivo* study of primary bronchial epithelial cells obtained from asthmatic children with or without wheeze that did not exhibit any viral or bacterial infection at the time of the sampling, showed that there is an association between the PI3K/Akt pathway and the integrin α5β1 expression with the epithelial cell migration, repair and healing (127). Recurrent wheeze in asthmatic children and exacerbation of asthma are associated with Dysregulation in the PI3K/Akt pathway (128). It is apparent that the delineation of the asthmatic pathophysiology along with its implication with resident microbiome will be promising for controlling, predicting and treating clinical asthma.

Polymeric immunoglobulin receptor (pIgR) on nasopharyngeal epithelium is a key mediator for *S. pneumoniae* serotypes adherence and translocation from the apical to the basolateral region of the cells and the subsequent release to the bloodstream (129, 130). *S. pneumoniae* counteracts antimicrobial agents secreted from the lungs by rejecting its capsule with the aid of autolytic enzyme LytA (131) and thus evading killing efficiently. The absence of pneumococcal capsule is not only a protective mechanism against cells but also a strategic plan for bacteria to better reach and interact with epithelial surfaces and promote infection and colonization (132). It is also well-established that genes translated to autolysin, pneumolysin, or hydrogen peroxide are significant virulence factors for *S. pneumoniae* to survive and cause infection events in the lungs (133–135). *S. aureus* is typically found in the nasal cavity, where it binds to mucosal epithelial surfaces and specifically to mucin (136, 137). Many adhesins present in bacterium surfaces like surface protein G (SasG) contributing to the high affinity of the species with the nasal cells (138, 139). Keratinized epithelial cells of nasal cavities play also a role in *S. aureus* adherence and colonization processes (140) through the binding of clumping factor B (ClfB) with cytokeratin type 10 (138). In addition, *S. aureus* can also adhere to non-mucosal surfaces via polysaccharides that can bind with fibronectin or collagen of extracellular matrix (141). Interestingly it has also been shown that *S. aureus* adheres better to nasal epithelium of patients with eczema compared to healthy controls (142). There is an unmet need to map the “behavior” of nasopharyngeal commensals on states of health and disease in order to better predict, intervene and cure chronic respiratory diseases.

Clinical studies have monitored the upper respiratory tract of various pediatric populations in order to identify the initial events of bacterial colonization. A number of surveillance studies on pediatric populations suggest that there are antagonistic relationship between the different serotypes of *S. pneumoniae and S. aureus* (143–149). In contrary, non-typeable *H. influenzae (*NTHi) and *M. catarrhalis* populations are strongly correlated with *S. pneumoniae* during their co-existence in the airways of healthy individuals (145, 146, 150, 151). It has been proposed that NTHi typically antagonizes *M. catarrhalis* colonization on nasopharyngeal mucosal surfaces as they share a few identical virulent factors (152). A mechanistic study, trying to shed light on the events influencing nasopharyngeal colonization in healthy children, looked into gene polymorphisms on their epithelial cell receptors (153). It was reported that healthy infants with TLR2 polymorphisms on their nasopharyngeal epithelial cells are more likely to be colonized by *S. aureus*, whereas infants with variants on mannose-binding lectin are simultaneously colonized by *S. aureus* and *S. pneumoniae*. Additionally, *M. catarrhalis* colonization rates are high in infants in which TLR2 and TLR4 polymorphisms were found together. Of note, the biological mechanisms and patterns, characterizing and influencing the co-colonization of respiratory pathogens, are not fully understood and are definitely affected overtime by the states of health and disease (94).

*In-vivo* experiments in mice report different outcomes related to the interactions of typeable or non-typeable strains of *H. influenzae* with *S. pneumoniae* during the co-colonization in the nasopharynx. NTHi and *S. pneumoniae* typically act with a common mechanism on epithelial surfaces as they both possess neuraminidase activity that enables the use of N-acetyl galactosamine β1-3 galactose on epithelial cells in order to bind and promote infection (154). It has been shown that initial infection with NTHi promotes in a synergistic way the secondary infection with *S. pneumoniae* in nasopharynxes of mice (155–157). In contrast, initial infection of murine nasopharynxes with a typeable serotype, results in a rapid clearance of subsequent *S. pneumoniae* addition (158). One possible explanation for this phenomenon is the activation by *H. influenzae* of a complement-dependent phagocytic mechanism that acts against *S. pneumoniae*. This study proposed that the innate immune system mediates and determines the competition between commensal bacteria.

Bacterial acquisition is influenced to a great extent by other resident bacteria dynamics (159). In the case of *S. aureus* it is proposed that accessory gene regulator (*agr*) and staphylococcal accessory regulator (*sar*) provoke cross-inhibition of the virulence factors expressed on respiratory pathogens (159). In addition, due to the fact that *S. aureus* is a stable population expressing high clonality compared to *S. pneumoniae* (160) we can assume that in the cases of a well-occupied nasopharyngeal niche with *S. aureus*, it is difficult for another antagonistic respiratory pathogen population to interfere. It is also well-known that *S. aureus* nasal carriers have more risk for *S. aureus* infections than infection from other bacteria (161). The above literature demonstrates complex interactions between epithelial cells and bacterial pathogens, when the abundances of the latter are influenced by constant changes and dysbiosis in the case of inflammatory conditions such as asthma.

#### Targeting Dysbiotic Bacteria With Bacteriophages

Recently, we have proposed the possibility of controlling dysbiotic bacterial populations in asthma through phage interventions (162). New therapeutic modalities for asthma could potentially include targeted and customized re-colonization of the upper airway with lytic phages against key bacteria, including the ability of phages to act as anti-biofilm agents (76). Isolation and characterization of naturally occurring phages against *M. catarrhalis, S. aureus, S. pneumoniae* and *H. influenzae* may have prominent importance in this context.

It is well-documented that co-evolution phenomena are taking place between phages and their respective hosts (163). Understanding the molecular and ecological characteristics of *M. catarrhalis* and its phages is an important target (164, 165). Until now, isolation of lytic phages for *M. catarrhalis* has not been achieved. Nevertheless, several prophages have been identified and characterized which could be indicative of the microbial ecology and evolution of the species and especially gram-negative bacteria that pre-occupy the respiratory niche as referred previously (166). Davie et al. described phage elements in a *M. catarrhalis* comparative genomic study of clinical isolates (167), while Ariff et al. also described the presence of at least 32 putative whole prophages harbored in the genomes of 95 *M. catarrhalis* clinical isolates (168). Although it is yet to be determined if these prophages could be active and able to infect the host if induced, these studies provide evidence of a large variety of prophages and prophage elements, which have interacted and fused their genome with *Moraxella* hosts over the years. Moreover, host molecular anti-phage strategies, such as CRISPR (169), against lytic phages had been well-documented, a strong indication of ancestral lytic phage-host interaction (167, 170). It is plausible that the large abundances of prophages and prophage elements (171) coupled with the significant presence of CRISPR systems harbored in *M. catarrhalis* genomes, could act as strong anti-phage strategies, making lytic phage isolation a challenging process.

*Haemophilus influenza*e is central in the basic phage research for many years (172–174). The binary models of *H. influenzae* and temperate phages HP1 and HP2 is a valuable tool for researchers to understand the lysis-lysogeny decision of temperate phages and the genes involved in it (175, 176). However, the literature still lacks a well-characterized virulent phage against this genus. Only recently researchers have reported a number of four possible isolates against *H. influenzae* with the complete characterization of these phages and their lytic efficacy remaining an open question (177).

Staphylococcal species colonize and dominate the human hypopharynx microbiome from early life (178). *S. aureus*has been suggested to be a serious threat for asthmatic incidents in adult life, through its superantigens (179). Contrary to *M. catarrhalis* and *H. influenzae*, staphylococcal lytic phages have been extensively studied as antimicrobial agents for many years against human and non-human bacterial infections. Systematic isolation and characterization of staphylococcal lytic phages has been taking place since 1950s. *S. aureus* was in the center of attention for the research community being a highly virulent agent with common antibiotic resistance, making it a high priority for discovering novel antimicrobial solutions against it. Interestingly, apart from the phages, lysozymes and endolysins were also tested against antibiotic-resistant strains of *S. aureus*, especially in strains colonizing skin tissue. Phage treatment has a much better background against *S. aureus* than other bacteria, offering promising results already (180, 181). Especially, Sb1 and ISP phages, expressing high virulence amongst antibiotic resistant *S. aureus* strains are used intravenously as therapeutics for many years now by the *Eliava* Institute of Bacteriophage, Microbiology and Virology (182, 183).

In regard to *S. pneumoniae*, lytic phages have also been isolated for many years now (184). *S. pneumoniae* demonstrates severe antibiotic resistance and apart from asthma-related incidents, it is a major causative agent for several respiratory diseases worldwide. Thus, novel approaches such as phage therapy could potentially have important role against it. Just as in *S. aureus*, potential therapeutic candidates are phage-originated murein hydrolases, which are able to degrade the outer thick peptidoglycan layer, the first line of defense for *gram*-positive bacteria, and induce lysis, without the use of phages (185–187).

### Bacteriophages and the Epithelium

#### Bacteriophages on Airway Mucosal Surfaces

Phages are part of the human virome; collectively, also referred as “phageome,” they regulate the populations of bacteria through prey-predator interactions (44). Recent studies show that phages are present in every niche of the human body including oral (188, 189), pulmonary (190, 191) and intestinal (192, 193) cavities and also the urinary tract (194), the skin (195, 196) and the blood (43, 197). The phageome constitutes the majority of viruses in a healthy lung and play a potentially crucial role to lung health and immunity, framing the bacterial population (45, 198). Bacteriophages have also been shown to reprogram the eukaryotic microbiome (199) and play an important role, as part of the immune system of mammals (200). The microbial load in healthy airways appears to be reduced as we proceed from the upper to the lower respiratory tract and except from the anatomical differences, it is also affected by the presence of different phage communities in different niches (26).

The surface of the nasal and bronchial epithelium is covered, as previously mentioned by mucus, while the surface of the alveolar epithelium is covered by pulmonary surfactant secreted from alveolar type II cells. Mucus contains mucin glycoproteins and nutrients that provide a favorable environment for commensal bacteria and phage symbionts. The most abundant types of mucin glycoproteins found in human airways are MUC5AC and MUC5B. *In-vivo* experiments in mice showed that MUC5B play a critical role in airway defense and bacterial clearance through MCC (30). *In-vitro* experiments with ALI differentiated bronchial epithelial cells from asthmatics, COPD and healthy control individuals, infected with rhinovirus-A1, showed no alteration of MUC5AC protein levels in any group (23). Nevertheless, the alteration of mucus patterns and production rates in many cases of clinical asthma provoke lung inflammation and multiple bacterial species colonization. In parallel, it is established that phage communities are more abundant in mucosal surfaces compared to adjacent non-mucosal areas (201, 202). The overall dysfunction of mucus secretions in asthma may influence not only the MCC mechanism but also the phage communities that regulate and control bacterial populations.

Mucus is an important intermediate in phage-bacteria interactions. In one related study they used T4 phages expressing or not an Ig-like protein (Hoc) in their capsid that binds to mucin glycoproteins. They performed *in-vitro* testing on mucus producing and mucus knockdown A549 lung epithelial cells. The cells were pretreated with hoc+ and hoc– T4 phages and then infected with *Escherichia coli* strains. They observed that mucus-producing cells pretreated with hoc+ phages, were protected from bacterial infection and cell death, in contrast to the mucus knockdown cells. In a microfluidic device simulating the constant air flow and mucin secretion dynamics, they showed the persistence of T4 phages and their subdiffusive motion on mucosal surfaces contributes to efficient phage-host encounters (201, 203). Although, recent literature about the interaction of phages with mucus is still limited, it is possible that future studies may unravel important mediation of these viruses in microbial equilibrium at the healthy airways.

During the last decade it was shown that several phages from the Myoviridae, Siphoviridae, and Podoviridae families, approximately one quarter of them, are carrying capsids with Ig-like proteins on their surfaces thus having the ability to complementary bind with mucin glycoproteins (204–206). In addition, it has been reported that there are large and diverse phage populations in mucosal surfaces compared with resident bacteria populations and with adjacent non-mucosal areas (201). We can assume that the direct association of phages with mucosal surfaces is providing a non-host-mediated mechanism of immunity (201) or an enhanced mechanism of host innate immunity that may induce a long-lasting adaptive immune response. In case of an infection, epithelial cells respond by secreting antimicrobial agents and mucin glycoproteins, thus giving the possibility for more phages to be attached and protect the surfaces from further bacterial colonization. The presence of phages in mucosal surfaces may also stimulate weak immune responses from the host thus inducing the continuous production of low concentrations of cytokines that may act protectively in the case of an infection (207, 208). Of note, it has been demonstrated *in-vitro* that Ig-like domains facilitate phage adsorption to the bacterial host and give a proliferation advantage against bacterial symbionts in mucosal areas (205, 206). It is becoming increasingly apparent that phages are key players for the overall homeostasis of human mucosal surfaces; further research may unravel new mechanisms and models for this complex symbiosis.

#### Bacteriophages and Epithelial Cell Interactions

The direct implication of phages with epithelial cells is a recently proposed concept, enhanced by several *in-vitro* studies. Lehti et al. propose that *E. coli* phage PK1A2, belonging to *Podoviridae* family, could directly interact with eukaryotic neuroblastoma epithelial cells. They used fluorescent and electron microscopy to observe the specific binding of phages with mammalian cells and follow the internalization process through the endolysosomal mechanism. Internalized phages remained intact for 24 h into the cytoplasm without affecting the viability of cells and then appeared in the lysosomes where their proteins and nucleic acids degraded from the cell (209). Recent reports, explain also the pivotal interactions that phages can develop with mammalian cells, including entering mammalian cells similarly to mammalian viruses, killing bacteria not only in the periplasmic space, but also both in the phagosome and the cytosol areas and finally express their genetic information in the nucleus (210). *In-vitro* experiments with bronchial primary epithelial cells, obtained from asthmatic children attempt to understand, as previously mentioned, the mechanisms of epithelial cell repairing and migration after wounding treatment (127). Future *in-vitro* and *in-vivo* studies could be focused on asthmatic epithelial repair during bacterial infection and/or phage presence. These hypotheses emphasize the critical role of phages in human environments and raise many questions about their undefined role, as protective, anti-inflammatory, biocontrol or nutrient agents for the cells.

Neuroblastoma cells express polysialic acid on their surfaces that shows high homology with polysaccharide of the bacterial host *E. coli* K1 of the phage. Additional molecules in airway epithelial surfaces have molecular homology with bacterial components, such as hyaluronan capsule of group A (211) and sialylated capsular polysaccharide of group B (212) *S. pneumoniae* and sialylated lipooligosaccharide of *Neisseria meningitides* (213). It appears that eukaryotic and prokaryotic cells exchange genetic material through HGT mainly after internalization of bacteria and/or phages in human cells. In addition, phages appear to have intrinsic tropism not only to bacterial cells but also to eukaryotic cells (214, 215). It is proposed that the molecular mimicry between bacteria and host cells may have an impact on phage's interaction with human cells.

Considering the enormous diversity of phage symbionts, a relevant number of epithelial epitopes may be capable to induce efficient intake of phages into the epithelial human cells. Specifically, it has been assumed that the human body can absorb ~10^10^ phage particles per day which can be transported through epithelial surfaces and reach the blood circulation (209, 216). Internalization and deposition of phages to the lymphatic and circulatory systems may also contribute to the remission of strong intracellular immunity signals that potentially make the cells resistant against microbial invaders (217). Typically, antigen recognition is a well-characterized ability of specific immune cells of the adaptive system; nevertheless Charles A. Janeway proposed that germline-encoded receptors on cells can sense bacterial conserved patterns and activate intracellular pathways for bacterial destruction after the cell invasion (218). We can assume that endocytosed phages in epithelial cells could be implicated in similar intracellular pathways that give the advantage for rapid response in the case of bacterial invasion.

#### Intra-Body Phages

Phages pass through epithelial mucosal surfaces via transcytosis (216), adhere to specific cell receptors and be endocytosed from the apical epithelial surface (215, 219). If they successfully escape lysosome degradation, phages are finally exocytosed from the basal side of the human cells and getting into the bloodstream from where they can reach distal areas (216, 220). This phenomenon was described for several phages such as T4, T5, T7, P22, SP01, and SPP1 (216). It was also reported that phages provided intraperitoneally or intranasally can be found even in the brain (221), suggesting the high motility and utterly different dynamic properties characterizing these viruses.

In an *in-vitro* study they used a series of different human epithelial cells from the colon (CaCo2 and T84), lung (A549), liver (Huh7), kidney (MDCK), and brain (hBMec) to monitor different phage families (*Podoviridae, Myoviridae, and Siphoviridae*) and to assess the ability of transcytosis across the confluent epithelial cell monolayers. Approximately 10% of epithelial cells expressed specific phage receptors and contained membrane-bound vesicles that allowed the internalization and transcytosis of phages (216). In another *in-vitro* study they used bovine mammary epithelial cells infected with *S. aureus* JYG2 in order to test the ability of virulent vB_SauM_JS25 phage to enter and lyse intracellular bacteria. After 12 h incubation with phages, they observed via confocal microscopy the presence of phages near the nuclei of infected cells and they established the progressive elimination of intracellular *S. aureus* in a time and dose dependent manner (222). In addition, It is reported that several phages such as Φ29, Bam35, Cp-1, Nf, PRD1 possess terminal proteins with inherent nuclear transmission signals (47) that enable the phage absorption to the eukaryotic nuclei and the effective delivery of proteins and genes into the eukaryotic genome (46). Transcytosis of phages enables the effective HGT between phages and eukaryotic cells and raise the question of whether endocytosed phages can replicate inside the cells and/or protect them from inflammation and cell death after bacterial invasion (210). Results from further experiments showed that phages can reduce inflammation in MAC-T bovine mammary epithelial cells suppressing the LPS-induced phosphorylation of NF-kB p65 protein from *S. aureus* (223).

The ability of phages to enter the cells and pass through internal organs is a new and yet not fully described phenomenon. The plethora of phages and the variety of human cells that can be used render the aforementioned experiments just the beginning for a new field of research. The characterization of the phenomenon demands the expansion of the related *in-vitro* and *in-vivo* studies in order to establish a good predictive model of the phage addition in the human body. Phages could play a significant role to host immunity and microbial balance and can be used as a novel therapeutic and anti-inflammatory tool in many chronic airway diseases. The future introduction of phages in clinical trials will be a new and promising step for the whole medical community and pharmaceutical industry, dealing with chronic and refractory respiratory diseases.

### Tripartite Interactions of Bacteria, Phages and the Airway Epithelium

#### Phages Develop Synergistic and/or Antagonistic Relationships With Bacteria on Airway Epithelial Surfaces Regulating Chronic Airway Inflammation

Although airway inflammation differs between asthma and cystic fibrosis (CF) important lessons can be learnt from the latter. Phage populations within the lungs of healthy individuals differ significantly compared with those suffering from CF (190). Different areas of explanted lungs of late-stage CF patients were examined with metagenomic analysis in order to identify and quantify different viral populations. They interestingly observed the absence of phages related to CF common pathogen *P. aeruginosa, S. aureus*, and *H. influenzae* at the left and right upper lobes (224), suggesting that phage communities play an important role in the homeostasis and overall health of the airways. Phage communities express similarities between CF patients and have a specific metabolic profile adapted to CF pathophysiology that differs significantly from the healthy state (225).

Recently, it has been reported that phage populations within the lungs of healthy individuals differ significantly compared with those suffering from asthma (162) and/or CF (190). The absence of phages related to common pathogens in asthma (162) and CF (224) suggests an important role of phage symbionts in airways homeostasis and healthy state. In an *in-vitro* study primary bronchial epithelial cells were isolated from children with CF and healthy controls and different concentrations of purified *P. aeruginosa* phage preparations were tested on submerged cell cultures in order to investigate the cytotoxic and/or inflammatory effect of phages. Results showed that the viability of cells was maintained, while no production of inflammatory cytokines IL-6 or IL-8 was observed. Of note, the apoptosis of cells was reduced in the presence of phages compared to untreated control cells (225). In contrast, *in-vivo* induction of filamentous phage Pf4 from *P. aeruginosa* biofilms enhances bacteria persistence in CF patient's lungs, promoting adhesion of the bacteria to mucus and inhibiting cell invasion (226). Therefore, *in-vivo* production of Pf4 phage leads to reduced lung injury, giving the fitness benefit to bacterial population to escape from phagocytosis and immune response. It is apparent that induction of prophage in this case changes the inflammation human response and lead to chronic persistence of bacterial population in CF lungs (227). From the aforementioned experiments we can expect that phages related to common pathogens in asthma may induce diverse behaviors *in-vitro* and *in-vivo*, favoring or eliminating bacterial populations.

Co-culture of *S. pneumoniae* with human cells can provoke the induction of streptococcal pyrogenic exotoxin C (SpeC), a well-characterized phage gene product. Interestingly this induction occurred without the addition of any phage induction reagents, like mytomycin C or application of UV radiation and just occurred during the co-culture. Broudy T. et al. examined the supernatants of the culture with electron microscopy to validate the existence of phage particles and they identified whole phage viruses during the co-culture of group A streptococci with Detroit 562 human pharyngeal cells (228). These results suggest the existence of a soluble yet uncharacterized phage inducing factor secreted from epithelial cells. This induction may occur independently from bacterial contact with human cells leading to the lysis of some streptococci and the subsequent release of cytoplasmic virulence factors. Subsequent *in-vitro* and *in-vivo* experiments in mice showed that the presence of mammalian cells may play a significant role in phage induction, implying that phages have obtained through natural selection a system where induction occurred at eukaryotic niches where bacteria are becoming more virulent (229).

The presence of prophage genetic elements with phage-encoded lysins in ~70% of clinically isolated *S. pneumoniae* strains (230) is a well-described phenomenon but the role of prophages during bacterial colonization is mainly undefined. In an *in-vivo* study, it was shown that the presence of Spn1 prophage element at *S. pneumoniae* provoked changes on bacterial cell wall and had a negative effect on nasopharyngeal colonization in mice (231). In another study, SV1 prophage elements were correlated with *S. pneumoniae* biofilm formation and promotion of chronic lung infection (232). Adherence of another nasopharyngeal human commensal *N. meningitides* to the epithelial surfaces was enhanced by the existence of filamentous prophage MDAΦ in an *in-vitro* study. This finding suggests that MDAΦ colonization of its host bacterium may increase occurrence of meningitis. Interestingly, this effect was not observed on endothelial cells, but only on epithelial cells tested (233).

In another *in-vitro* study, phiCDHS1 lytic phages appeared more active against *Clostridium difficile* in the presence of HT-29 epithelial cells than without them. They performed subsequent experiments to audit whether there are specific HT-29 secretions that enhance phage activity, culturing phages with fresh or spend HT-29 culture medium. They concluded that phage lysis activity is not affected by the substances on cell culture media but it is promoted by the adherence of phages to the epithelial monolayer facilitating subsequent adherence with *C. difficile* (234).

An interesting example of nasopharyngeal microbiome regulation by phages has been reported regarding *S. pneumoniae* and *S. aureus* interactions. It was reported that *S. aureus* may be restricted from the nasopharynx in the presence of *S. pneumoniae* (235). H_2_O_2_ produced by *S. pneumoniae* promotes the production of hyperoxides via Fenton chemical reaction. Hyperoxides are able to induce SOS response in *S. aureus* that is typically a reaction that promotes DNA restoration, causing the activation of resident prophages from *S. aureus* lysogenic strains. Subsequent lysis of *S. aureus* promotes the *S. pneumoniae* prevalence at the nasopharynx. In contrary, *S. pneumoniae* lysogenic strains do not undergo SOS-response thus having a prevalence asset against *S. aureus* symbionts. The induction of prophages in neighboring organisms is a strategy for releasing active phages into the human body. This effect can cause subsequent spread of virulence genes and mobile genetic elements into different cells thus reshaping the existent equilibrium (236).

**Figure 1:**
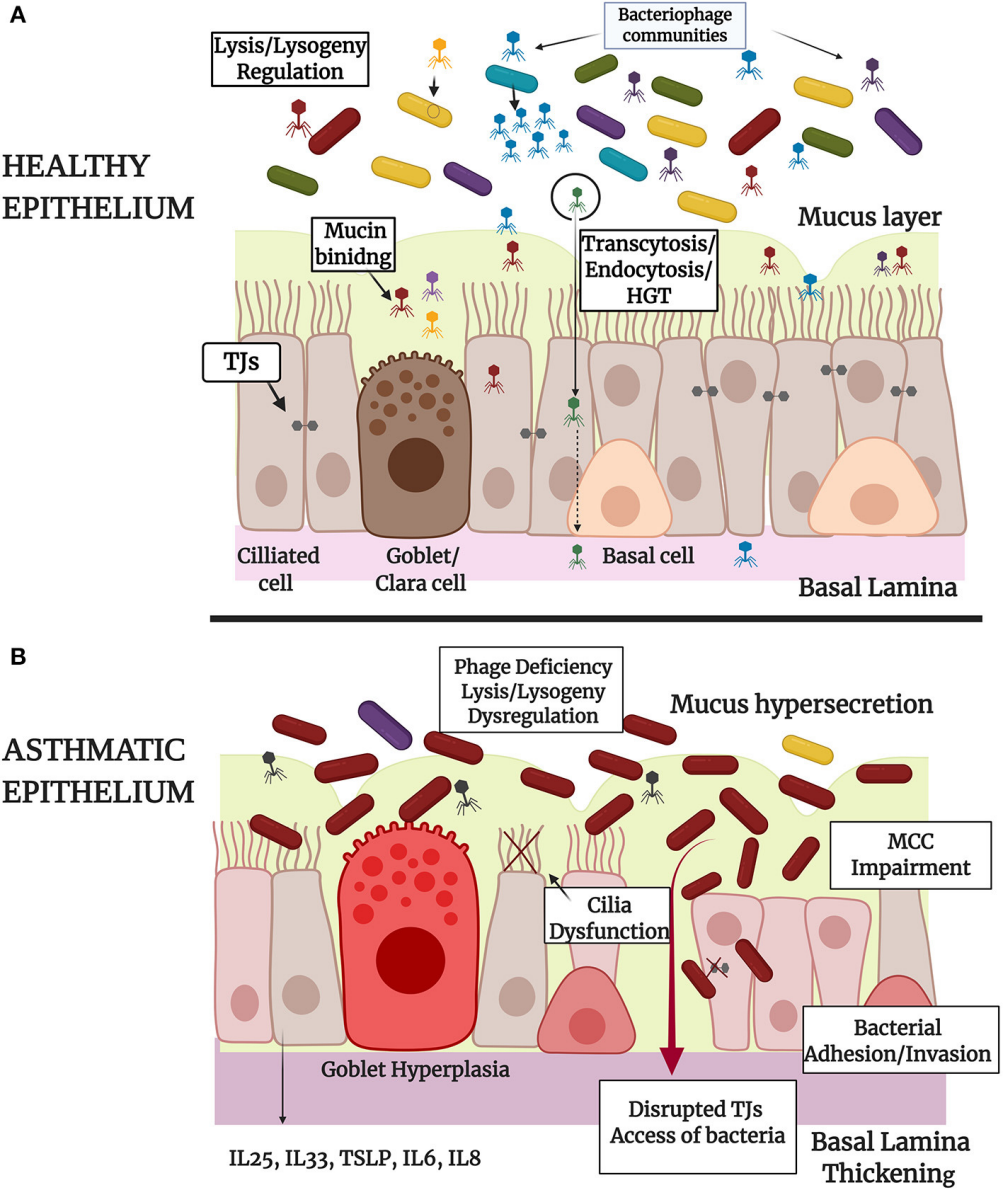
Schematic representation of the tripartite symbiosis model between bacteriophages, bacteria and epithelium within healthy **(A)** and asthmatic **(B)** airways. **(A)** Healthy epithelium of large and small airways of the lungs is constituted by basal, ciliated and secretory Goblet (large airways) or Clara cells (small airways) that form pseudostratified or columnar and cuboidal structures, respectively. Epithelial barrier integrity is ensured by the formation of intracellular Tight Junctions (TJs) and by the secretion of mucus by Goblet or Clara cells. Healthy epithelium physically controls bacterial populations and eliminates bacterial invasion through mucociliary clearance (MCC). In healthy airways there is a diverse population of bacteria and bacteriophage communities that interact directly through lysis-lysogeny mechanisms and horizontal gene transfer as represented in the figure. Bacteriophages interact also directly with epithelium through adhesion to mucus or through specifically biding to cellular receptors (endocytosis) and non-specific binding to membrane vesicles (transcytosis). Bacteriophage-epithelium uptake protects the cells from bacterial invasion and proliferation and enables the delivery of bacteriophage nucleic acids (HGT) and proteins into the nucleus. **(B)** Asthmatic epithelium is characterized by Goblet hyperplasia, mucus hypersecretion, MCC impairment and disrupted TJs that lead to bacterial adherence and invasion into the human cells or access of bacteria into the lamina paracellularly. In the case of asthma, bacteriophage communities are deregulated, as it is presented in the figure, and this may be associated with an altered microbiota profile with limited bacterial diversity and dominance of specific bacterial species. The role of prophage elements in bacterial genomes has also an influence on the ability of pathogens to provoke infection and epithelial damage. Irritated asthmatic epithelium secretes a range of chemokines and cytokines, contributing to immune system activation and chronic inflammation in asthma. The image was created using BioRender.

From the aforementioned literature it is clear that phages possess high immune modulatory capacities (207, 237) in CF (238, 239), COPD (240, 241), and other respiratory disease conditions. To the extent of our knowledge there are limited relevant published studies about the role that phages own on asthmatic epithelium. It cannot be excluded that microbial imbalance in asthma may be related and/or caused by underlying phage imbalance (162). In the near future, with the continuous research on that field, we expect the establishment of new mechanisms of phages-bacteria-eukaryote cells interactions in order to better orchestrate the triptych symbioses model (Figure 1).

## Discussion

Current literature related to the triptych bacteria–phages–airway epithelium highlights a number of aspects of this dynamic system. The new era of precision medicine demands the construction of a well-established system for personalized monitoring (28). Emerging molecular and screening techniques could give the possibility to stratify the microbiome of an individual from infancy until adulthood. In this way we may be able to map dynamic microbiome profiles thus following and understanding the alterations occurring during an infection or in the onset of a chronic disease. Novel lytic phages could play a pivotal role in restoring bacteria pathogens abundances, thus making their isolation and characterization a priority challenge. Still, literature lacks well-characterized lytic phages for important bacterial species that colonize the human hypopharynx, such as *M. catarrhalis* and *H. influenzae*.

Researchers have already pinpointed the importance of studying the biology of the wider system when phage therapy is taking place, rather than only the binary interaction of bacterial host and virus (242–244). The incidences of phage-bacteria interaction on epithelial surfaces are influenced to a great extent by the subdiffusive motion of phages on mucosal surfaces (203). In addition, another important parameter is the motility rate of bacterial hosts on specific environments where phages can be added. Specifically high bacterial motility rates are related with more frequent phage encountering independently of the phage motion in the mucus (244). We must also take into account the ability of some bacteria to form biofilms on human airway niches and thus further evaluate the anti-biofilm capacity of important lytic phages (245, 246).

There is still limited number of studies on phage-bacteria population interactions *in-vivo*. The existent studies are using mainly mice models in order to predict and evaluate the effect of phage addition on the gut microbiome (247–249). The potential changes related with phage intervention on the airways are mainly unexplored. The addition of lytic phages on the mouse gut can affect not only the sensitive bacteria but also other resident bacteria populations in a cascade (249). It is thus important to attempt to predict the new dynamics that will be created following potential phage therapy at the respiratory tract.

In this review, we attempt to shed light to a new field of basic research, focused on the tripartite interactions between respiratory epithelial cells, bacteria and phages and to connect its results with asthma pathophysiology. Concepts emerging through this review need to be tested in the clinic. In Table 2 we list a number of such concepts, suggesting targets for future clinical studies.

**Table 2:** Novel ideas that are coming up from current *in-vitro* studies focused on the interaction between bacteriophages, bacteria and airway mucosa and proposed future *in-vivo* studies that can be significant for clinical asthma.

**References**	**Interaction entities**	**Purpose of *in-vitro* studies**	**Focus of future clinical studies**
Tosi (103); Smith-Vaughan et al. (102); Spaniol et al. (115); de Vries et al. (111); Post et al. (112)	Bacteria-Epithelial cells	Study bacterial adhesion and invasion events at epithelial cells	Understand the antagonistic and/or synergistic relationships between airway bacteria during colonization of the airways
Aalto et al. (214); Dabrowska et al. (215); Tam and Jacques (217); Lehti et al. (209); Nguyen et al. (216); Bodner et al. (210); Iosifidis et al. (127)	Bacteriophage-Epithelial cells	Understand how internalized phages may act as protective, anti-inflammatory, biocontrol or nutrient agents for the cells	Study the effect of bacteriophage interventions on asthmatic airways
Barr et al. (201); Meyer (202); Dickson et al. (26)	Bacteriophages-Airway mucosa	Understand how alteration of mucus consistency can influence bacteriophage communities	Characterize bacteriophage communities on mucus secretions from asthmatic and healthy individuals
Fraser et al. (205, 206); Barr et al. (201, 203); Meyer (202)	Bacteriophages-Airway mucosa-Bacteria	Understand how bacteriophages attached to mucus can protect epithelial cells from bacterial infection	Understand how airway mucosal surfaces and bacteriophages act synergistically during bacterial infection in healthy individuals
Duerkop and Hooper (207); Sinha and Maurice (200); Federici et al. (199)	Bacteriophages-Airway mucosa	Understand how bacteriophages attached to mucus may stimulate weak immune responses protecting from possible infection	Serological studies of the antibodies produced against bacteriophages attached on mucosal surfaces of healthy individuals
Selva et al. (236); Tam and Jacques (217); Bodner et al. (210)	Bacteriophages- Epithelial cells-Bacteria	Understand how bacteriophages regulate bacterial populations in the presence of respiratory epithelial cells	Intervene with bacteriophages in order to control dysbiotic bacteria in the airways of asthmatic patients

## Author Contributions

PT-T designed and wrote the manuscript. DS contributed textually and designed along with PT-T the Figure. NP and SM conceptualized the main idea. NP coordinated the whole review. All authors contributed critically on reviewing and making substantial comments on the final article.

## Conflict of Interest

The authors declare that the research was conducted in the absence of any commercial or financial relationships that could be construed as a potential conflict of interest.
