# Corrigendum: Interactions of Bacteriophages and Bacteria at the Airway Mucosa: New Insights Into the Pathophysiology of Asthma

**DOI:** 10.3389/falgy.2022.892908

**Published:** 2022-04-01

**Authors:** Panagiota Tzani-Tzanopoulou, Dimitrios Skliros, Spyridon Megremis, Paraskevi Xepapadaki, Evangelos Andreakos, Nina Chanishvili, Emmanouil Flemetakis, Grigoris Kaltsas, Styliani Taka, Evangelia Lebessi, Anastassios Doudoulakakis, Nikolaos G. Papadopoulos

**Affiliations:** ^1^Allergy and Clinical Immunology Unit, Second Pediatric Clinic, National and Kapodistrian University of Athens, Athens, Greece; ^2^Laboratory of Molecular Biology, Department of Biotechnology, School of Food, Biotechnology and Development, Agricultural University of Athens, Athens, Greece; ^3^Division of Evolution and Genomic Sciences, University of Manchester, Manchester, United Kingdom; ^4^Center for Clinical, Experimental Surgery and Translational Research of the Biomedical Research Foundation of the Academy of Athens, Athens, Greece; ^5^Laboratory for Genetics of Microorganisms and Bacteriophages, Eliava Institute of Bacteriophage, Microbiology & Virology, Tbilisi, Georgia; ^6^Department of Electrical and Electronic Engineering, University of West Attica, Athens, Greece; ^7^Department of Microbiology, P. & A. Kyriakou Children's Hospital, Athens, Greece

**Keywords:** bacteria, asthma, bacteriophages, airway mucosa, tripartite symbiosis

**Incorrect Affiliation**

In the published article, there was an error in affiliation **Number 5**. Instead of “**Laboratory for Genetics of Microorganisms and Bacteriophages, Eliava Institute of Bacteriophage, Microbiology & Virology, Tbilisi, GA, United States**,” it should be “**Laboratory for Genetics of Microorganisms and Bacteriophages, Eliava Institute of Bacteriophage, Microbiology & Virology, Tbilisi, Georgia**.”

The authors apologize for this error and state that this does not change the scientific conclusions of the article in any way. The original article has been updated.

## Publisher's Note

All claims expressed in this article are solely those of the authors and do not necessarily represent those of their affiliated organizations, or those of the publisher, the editors and the reviewers. Any product that may be evaluated in this article, or claim that may be made by its manufacturer, is not guaranteed or endorsed by the publisher.

